# Quality of First Prenatal Consultations in Malemba Nkulu, Democratic Republic of Congo: Challenges and Opportunities in a Cross-Sectional Study

**DOI:** 10.2196/78511

**Published:** 2025-10-01

**Authors:** Fiston Ilunga Mbayo, Pascal Geri Madragule, Pacifique Kanku wa Ilunga, Ignace Bwana Kangulu, Dalau Nkamba Mukadi

**Affiliations:** 1 School of Public Health Faculty of Medicine University of Kinshasa Kinshasa the Democratic Republic of the Congo; 2 Department of Public Health General Referral Hospital of Malemba Nkulu Malemba Nkulu the Democratic Republic of the Congo; 3 Faculty of Medicine University of Kamina Kamina the Democratic Republic of the Congo

**Keywords:** quality, antenatal, consultation, maternal health, Democratic Republic of Congo

## Abstract

**Background:**

Maternal mortality remains alarmingly high in the Democratic Republic of Congo (DRC), particularly in rural areas where access to quality prenatal care is limited. Despite global efforts to improve maternal health, systemic gaps persist in the delivery of antenatal services.

**Objective:**

The objective of this study is to assess the quality of first antenatal consultations in the Malemba Nkulu health zone and identify structural and procedural factors contributing to substandard care.

**Methods:**

A cross-sectional descriptive study was conducted in November 2023 across 8 health facilities selected through simple random sampling. Data were collected from 248 pregnant women attending their first prenatal visit and from 14 health care providers. Quality indicators were assessed using a structured checklist based on World Health Organization (WHO) standards. Variables included provider qualifications, availability of diagnostic tools, and completeness of clinical assessments.

**Results:**

Only 2% (5/248) of first antenatal consultations met the minimum quality standards. Major deficiencies included lack of physical examinations 78% (193/248), absence of essential laboratory tests 92% (228/248), and inadequate counseling 85% (212/248). Facilities lacked basic equipment such as blood pressure monitors and hemoglobin tests. Provider training was inconsistent, and community awareness of prenatal care remained low.

**Conclusions:**

The quality of first antenatal consultations in Malemba Nkulu is critically poor, reflecting broader systemic challenges in rural maternal health care. Strengthening provider training, improving infrastructure, and enhancing community engagement are essential to reduce maternal mortality and improve outcomes in resource-limited settings.

## Introduction

### Background

Maternal mortality remains a major public health concern in low-resource settings, particularly in sub-Saharan Africa. According to the World Health Organization (WHO), nearly 99% of maternal deaths occur in countries with limited access to quality care [1]. In the Democratic Republic of Congo (DRC), the maternal mortality ratio is estimated at 473 deaths per 100,000 live births [2], a figure that reflects persistent gaps in the health system and the urgent need for targeted interventions.

Antenatal care (ANC) is widely recognized as a key strategy to reduce maternal and neonatal morbidity and mortality. The WHO recommends at least 8 prenatal visits, with the first ideally occurring before 12 weeks of gestation [3]. Early and high-quality ANC enables timely screening, prevention of complications, and health education. However, in the DRC, fewer than 8% of pregnant women initiate ANC in the first trimester [4], and many consultations are conducted by providers lacking formal medical training [5].

The Malemba Nkulu health zone, located in the rural province of Haut-Lomami, exemplifies these challenges. It was selected for this study due to its high maternal risk profile, limited access to qualified health personnel, and absence of recent data on ANC quality. Preliminary data from this region reveal that over half of pregnant women (52.4%) attend their first ANC visit in the third trimester, limiting the effectiveness of preventive measures. Additionally, nearly 43% of consultations are conducted by traditional birth attendants without advanced medical qualifications, and more than 60% of health facilities lack essential equipment for complete prenatal assessments, including tests for anemia and maternal infections [6]. These findings suggest that the quality of the first antenatal consultation (ANC1) may be a critical determinant of maternal outcomes. While previous studies in West Africa have linked ANC quality to provider training, infrastructure, and community engagement [7], few have examined this issue in depth within the DRC. Evidence from Rwanda and Ethiopia shows that integrating ANC into community health programs and investing in provider capacity can significantly improve maternal care [8].

### Objective

This study aims to assess the quality of the first antenatal consultation in the Malemba Nkulu health zone using national and international standards as benchmarks. It seeks to identify structural, procedural, and contextual factors that influence care quality and to inform strategies for improving maternal health in rural Congolese settings.

## Methods

### Study Design and Setting

This study used a descriptive cross-sectional design to assess the quality of ANC1s in the Malemba Nkulu health zone. The study was conducted over a 2-month period from November to December 2023. This design was selected to provide a snapshot of service delivery and identify gaps in compliance with national and WHO standards.

### Study Site

This study took place in the Malemba Nkulu health zone, located in the rural province of Haut-Lomami in southeastern DRC. The area is characterized by limited access to qualified health personnel, inadequate infrastructure, and high maternal risk indicators, making it a relevant setting for evaluating ANC quality.

### Sampling and Methodological Justification

This study used a mixed sampling approach, combining probabilistic and nonprobabilistic techniques to ensure both representativeness and feasibility in a resource-limited rural context.

#### Health Facilities

Eight facilities were selected using simple random sampling from the official list of centers providing ANC services within the Malemba Nkulu health zone. This method ensured equal probability of selection and minimized selection bias.

#### Pregnant Women

A total of 248 pregnant women were recruited through consecutive sampling, including all eligible participants attending their ANC1 during the study period. This approach captured real-time practices without interruption or subjective filtering.

#### Health Care Providers

Fourteen providers were included through exhaustive sampling, meaning all active personnel involved in ANC1 services at the selected facilities were enrolled, provided they had at least 6 months of experience in antenatal care. This ensured a comprehensive representation of professional profiles and practices.

#### Methodological Rationale

This sampling strategy was chosen to maximize data representativeness in a heterogeneous rural environment, minimize bias related to participant availability and provider selection, and facilitate logistical implementation under field conditions.

### Study Population and Eligibility Criteria

#### Pregnant Women

The inclusion criteria for pregnant women included those (1) attending their ANC1 during the study period, (2) receiving care in one of the selected facilities, and (3) providing informed consent after explanation of study objectives. Pregnant women were excluded if their ANC1 was previously conducted in another facility or if they refused to participate.

#### Health Care Providers

The inclusion criteria for health care providers included those (1) actively involved in delivering ANC services in the selected facilities and (2) with a minimum of 6 months’ experience in antenatal care provision. Health care providers were excluded if they were absent during the study period due to leave or reassignment or refused to participate.

#### Sampling Strategy and Justification

Health facilities were selected using simple random sampling to ensure equal probability of inclusion and reduce selection bias. Pregnant women were recruited consecutively, including all eligible women presenting for ANC1 during the study period. Health care providers were included exhaustively from the selected facilities, based on availability and direct involvement in ANC.

This mixed sampling approach was chosen to maximize representativeness and feasibility. The final sample included 248 pregnant women and 14 health care providers, reflecting the diversity of practices and qualifications across the zone.

### Data Collection Procedures

Data were collected using a standardized observation grid adapted from national guidelines and WHO recommendations. Ten key dimensions of ANC1 were assessed, as outlined in Table 1.

Each consultation was directly observed by trained data collectors. Medical records were reviewed to confirm procedures, and structured interviews were conducted with pregnant women to assess their perceptions of care.

**Table 1:** A summary for the measurement of the first antenatal consultation (ANC1) quality variable. The maximum total ANC1 quality score is 100, with each domain contributing up to 10 points.

Domains and their objects			Measure		Percentage rating per object			
**History of pregnant women**								
	Age of the pregnant woman	1=Requested, 0=No		2.5				
	Medications that the pregnant woman takes	1=Requested, 0=No		2.5				
	Date of start of the pregnant woman’s last menstrual period	1=Requested, 0=No		2.5				
	Number of previous pregnancies the pregnant woman has had	1=Requested, 0=No		2.5				
**Aspects of previous pregnancies**								
	Previous stillbirth(s)	1=Requested, 0=No		1				
	Infant(s) who died during the first week of life	1=Requested, 0=No		1				
	Heavy bleeding during or after childbirth	1=Requested, 0=No		1				
	Previous assisted delivery (eg, cesarean, vacuum, or forceps)	1=Requested, 0=No		1				
	Previous spontaneous abortions	1=Requested, 0=No		1				
	Previous multiple pregnancies	1=Requested, 0=No		1				
	Previous prolonged work	1=Requested, 0=No		1				
	Hypertension induced by previous pregnancy	1=Requested, 0=No		1				
	Seizures related to a previous pregnancy	1=Requested, 0=No		1				
	High fever or infection in previous pregnancies	1=Requested, 0=No		1				
**Danger signs of ongoing pregnancy**								
	Vaginal bleeding	1=Requested, 0=No		1.43				
	Fever	1=Requested, 0=No		1.43				
	Headache or blurred vision	1=Requested, 0=No		1.43				
	Swollen face, hands, or extremities	1=Requested, 0=No		1.43				
	Fatigue or shortness of breath	1=Requested, 0=No		1.43				
	Cough or difficulty breathing for 3 weeks or more	1=Requested, 0=No		1.43				
	Any other symptoms or problems related to this pregnancy	1=Requested, 0=No		1.43				
**Physical examination**								
	Taking the pregnant woman’s blood pressure	1=Executed, 0=No		1.25				
	Weighing the pregnant woman	1=Executed, 0=No		1.25				
	Examine the conjunctiva or palms for anemia	1=Executed, 0=No		1.25				
	Examine legs, feet, and hands for edema	1=Executed, 0=No		1.25				
	Examine any swollen lymph nodes or glands	1=Executed, 0=No		1.25				
	Palpate or measure the pregnant woman’s abdomen for fundal height	1=Executed, 0=No		1.25				
	Examine the pregnant woman’s breasts	1=Executed, 0=No		1.25				
	Perform a vaginal examination or perineal examination	1=Executed, 0=No		1.25				
**Routine examinations**								
	Hemoglobin test	1=Executed, 0=No		2.5				
	Blood typing	1=Executed, 0=No		2.5				
	Any urine test	1=Executed, 0=No		2.5				
	Syphilis test	1=Executed, 0=No		2.5				
**HIV testing and counseling**								
	Asked if the pregnant woman knew her HIV status	1=Requested, 0=No		2				
	Provide or refer for advice related to HIV testing	1=Executed, 0=No		2				
	Perform or refer for an HIV test	1=Executed, 0=No		2				
	Providing posttest counseling	1=Executed, 0=No		2				
	Discussion on partner testing	1=Discussed, 0=No		2				
**Maintaining a healthy pregnancy**								
	Discussed nutrition (ie, food to eat) during pregnancy	1=Discussed, 0=No		3.33				
	Informing the pregnant woman about the progress of the pregnancy	1=Executed, 0=No		3.33				
	Discussed the importance of at least 4 prenatal visits	1=Executed, 0=No		3.33				
**Iron and folate supplementation**								
	Prescribed or given iron or folic acid tablets or both	1=Executed, 0=No		2.5				
	Explained the purpose of iron or folic acid	1=Executed, 0=No		2.5				
	Explained how to take iron or folic acid tablets	1=Executed, 0=No		2.5				
	Explanation of the side effects of iron or folic acid tablets	1=Executed, 0=No		2.5				
**Tetanus toxoid injection**								
	Prescribed or administered a TAT^a^ injection	1=Executed, 0=No		3.33				
	Explained the purpose of TAT injection	1=Executed, 0=No		3.33				
	TAT card verified or ANC^b^ card	1=Executed, 0=No		3.33				
**Preparation for childbirth**								
	Asked the pregnant woman where she will deliver	1=Requested, 0=No		2				
	Advised the pregnant woman to prepare for delivery	1=Recommended, 0=No		2				
	Advised the pregnant woman to seek the assistance of a qualified health worker for delivery	1=Recommended, 0=No		2				
	Indicate what items to have on hand in case of emergency	1=Recommended, 0=No		2				
	Advised the pregnant woman to give birth in a health facility	1=Recommended, 0=No		2				

^a^TAT: tetanus toxoid.

^b^ANC: antenatal care.

### Variables

Independent variables included the following: (1) type of health facility (health center or general hospital), (2) provider profile (level of qualification and professional experience), (3) patient characteristics (age, education level, parity, and trimester of pregnancy at the time of ANC1), (4) availability of resources (medical equipment and access to laboratory tests), and (5) accessibility of care (distance from home to facility and consultation cost).

Dependent variables included the following: (1) overall conformity of the consultation (compliant or noncompliant), (2) completion of clinical and laboratory examinations, (3) quality of information provided to the patient (nutrition, danger signs, and birth planning), (4) preventive and curative interventions (prescription of iron or folic acid, tetanus vaccination, and malaria treatment), and (5) patient satisfaction (reception, waiting time, cost, and quality of counseling received).

### Handling of Missing Data

Missing data were identified through systematic review of observation grids and medical records. Completely missing data were excluded from the analysis, and partially missing data were imputed using weighted averages and predictive modeling. Sensitivity analyses were conducted to assess the impact of missing data on key indicators.

### Data Analysis

Data were entered and analyzed using SPSS version 25.0 (IBM Corporation). Descriptive statistics were used to calculate frequencies, proportions, means, and SDs. The results were stratified by facility type and provider qualification, and 95% CIs were calculated for key indicators. *P* values were reported where applicable, with statistical significance set at *P*<.05.

### Ethical Considerations

This study was conducted in full compliance with the ethical principles outlined in the Declaration of Helsinki and the guidelines of the National Ethics Committee of the Democratic Republic of Congo. Ethical approval was obtained from the Ethics Committee of the School of Public Health at the University of Kinshasa, under reference number ESP/CE/099/2023, in accordance with institutional procedures.

Before any data collection, written informed consent was obtained from each pregnant woman included in the study, following a clear explanation of the study’s objectives and participation procedures. Participation was entirely voluntary, with no form of pressure or financial or material incentive.

Data confidentiality was ensured through anonymized records and the use of secure identification codes. All data were stored in protected files, accessible only to authorized members of the research team.

The authors declare no conflicts of interest related to this research. The study is part of a broader effort to strengthen local maternal public health capacities and to promote medical practices in rural settings.

## Results

The distribution of pregnant women by the quality of the ANC1, according to the criteria defined by national and international standards, is shown in Table 2. It shows the percentage of consultations that meet quality standards and highlights gaps in care.

**Table 2:** Distribution of pregnant women according to the quality of the first prenatal consultation (ANC1) received (N=248).

Quality of ANC1	Pregnant women, n (%)	95% CI
Good quality	5 (2)	1.86-2.16
Poor quality	243 (98)	3.51-5.36

The study found that 61% (151/248) of health facilities lacked the necessary equipment to perform a comprehensive prenatal checkup. Essential laboratory tests such as syphilis screening and anemia assessment were systematically absent in 45% (112/248) of cases. Table 3 illustrates the availability and performance of essential laboratory tests at the first antenatal visit. It highlights gaps in screening, including the absence of hemoglobin tests, blood typing, and syphilis screening.

A significant proportion of consultations did not include all of the recommended examinations or adequate advice on pregnancy management.

Our analysis highlighted several constraints affecting the quality of ANC1. Among the limiting factors, 43% (107/248) of consultations were conducted by midwives without advanced medical training. Furthermore, 52% (129/248) of women had their first ANC in the third trimester, which significantly reduces the impact of preventive measures.

Table 4 describes the characteristics of the prenatal care providers who performed the consultations. It highlights their education level, average age, and sex distribution. Notably, a majority of providers did not have advanced medical training.

**Table 3:** Assessment of the quality of prenatal consultations according to laboratory tests.

Laboratory tests received		Pregnant women (N=248), n (%)
**Hemoglobin test**		
	No	215 (86.7)
	Yes	33 (13.3)
**Blood typing**		
	No	240 (96.8)
	Yes	7 (2.8)
**Urine test**		
	No	218 (87.9)
	Yes	30 (12.1)
**Syphilis test**		
	No	243 (98)
	Yes	5 (2)

**Table 4:** Distribution of prenatal care providers according to their sociodemographic characteristics.

Features		Providers (N=14)
Age (years), mean (SD)		49.3 **(**6)
**Educational level, n (%)**		
	Nurse A2	8 (57.1)
	None	6 (42.9)
**Sex, n (%)**		
	Female	14 (100)
	Male	0 (0)

Analysis of interactions between providers and pregnant women showed that only 37% (149/248) of consultations included in-depth education on nutrition, danger signs, and childbirth preparation. The average consultation duration was less than 15 (SD 3) minutes in 68% (169/248) of cases, limiting the quality of support.

Table 5 summarizes pregnant women’s opinions on various aspects of their first prenatal visit, including cost, distance, reception by health workers, and waiting time. It provides insight into pregnant women’s perceptions of the quality of care received.

**Table 5:** Assessment of the quality of prenatal consultation on the judgment of pregnant women.

Judgement		Pregnant women (N=248), n (%)
**Cost**		
	Affordable	100 (40.3)
	Pupil	114 (46)
	Lower	22 (8.9)
	Higher	12 (4.8)
**Distance**		
	Short	59 (23.8)
	Long	66 (26.6)
	Normal	88 (35.5)
	Too short	24 (9.7)
	Too long	11 (4.4)
**Welcoming health workers during ANC1** ^a^		
	Not satisfactory	40 (16.1)
	Satisfying	158 (63.7)
	Very satisfactory	50 (20.2)
**Waiting times for prenatal consultations**		
	Long	138 (55.6)
	Not long	97 (39.1)
	Too long	13 (5.2)

^a^ANC1: first antenatal consultation.

Less than 50% (124/248) of women received necessary nutritional supplements, such as iron and folic acid. Vaccination coverage and the administration of antimalarial drugs were also limited, compromising the prevention of maternal and neonatal complications.

Table 6 details the elements covered during the prenatal consultation related to maternal and newborn health. It analyzes nutrition awareness, the importance of prenatal monitoring, the prescription of nutritional supplements and vaccines, as well as information on pregnancy management.

**Table 6:** Assessment of the quality of prenatal consultation on maintaining a healthy pregnancy.

Aspects discussed for maintaining a healthy pregnancy		Pregnant women (N=248), n (%)
**Nutrition discussion**		
	No	93 (37.5)
	Yes	155 (62.5)
**Information on the progress of the pregnancy**		
	No	95 (38.3)
	Yes	152 (61.3)
**Discussion of the importance of at least 4 prenatal visits**		
	No	93 (37.5)
	Yes	155 (62.5)
**Prescription of iron tablets**		
	No	36 (14.5)
	Yes	212 (85.5)
**Explanation of the purpose of iron**		
	No	217 (87.5)
	Yes	31 (12.5)
**Explanation of how to take iron**		
	No	40 (16.1)
	Yes	208 (83.9)
**Prescription or injection of tetanus vaccines**		
	No	34 (13.7)
	Yes	214 (86.3)
**Explanation of the purpose of the tetanus vaccine injection**		
	No	68 (27.4)
	Yes	180 (72.6)

## Discussion

### Principal Findings

The findings of this study revealed that only 2% of ANC1s met minimum quality standards in the Malemba Nkulu health zone. This reflects broader systemic challenges in rural maternal health care, including limited provider training, lack of diagnostic tools, and delayed access to care.

Similar challenges have been documented in other Central and West African contexts. In Lubumbashi, DRC, a study found that only 0.2% of pregnant women initiated ANC in the first trimester, and most consultations were conducted by nurses with limited qualifications [9]. In Mali, research conducted in Sélingué highlighted gaps in counseling and continuity of care, despite satisfactory physical examinations and reception [10]. Another study in Bougouni emphasized the importance of integrating ANC into reproductive health programs and strengthening provider training [11].

These regional findings reinforce the relevance of our results and suggest that targeted interventions—such as community-based outreach, provider certification, and subsidized diagnostic services—could improve ANC quality across similar settings. They also support evidence from Rwanda and Ethiopia, where continuous training and integration of ANC into community health systems have led to measurable improvements in maternal outcomes [12,13].

The results obtained in this study are consistent with those reported in other research in sub-Saharan Africa. For example, a study conducted in Nigeria showed that more than 60% of antenatal consultations were carried out by unqualified personnel, leading to insufficient follow-up [14]. Research in Ghana has shown a direct link between the quality of antenatal consultations and obstetric complication rates [15]. Additionally, in Rwanda and Ethiopia, the quality of antenatal care has improved considerably thanks to policies targeting continuous training of providers and integration of antenatal services into community care [12,13]. These successes suggest that adapted strategies could be implemented in the DRC to improve the effectiveness of prenatal consultations and reduce maternal morbidity [16].

### Factors Influencing the Quality of Care

Among the main causes of the insufficient quality of prenatal consultations in the health zone studied, the following elements stand out:

1. Training of providers: 43% (107/248) of consultations were carried out by midwives without advanced medical training, which limits the application of standardized protocols [17].
2. Late access to care: 52% (129/248) of women had their first prenatal consultation in the third trimester, thus reducing the impact of preventive interventions [18].
3. Lack of medical equipment: 61% (151/248) of health facilities did not have the necessary tools to carry out a complete prenatal assessment, compromising the quality of monitoring [19].
4. Cost of services: the high cost of consultations and additional examinations is a barrier to pregnant women’s adherence to medical recommendations [16].

### Implications for Health Policy

These results highlight the urgency of implementing strategies to strengthen the quality of prenatal care. Among the possible measures, several recommendations can be made, including:

1. Strengthening the training of providers, particularly through practical workshops and certifications in prenatal monitoring [12,13].
2. Subsidy of prenatal services to improve accessibility to care [14,15].
3. Community awareness to encourage pregnant women to consult from the first trimester [17].
4. Integration of prenatal care into maternal health programs to ensure comprehensive and effective monitoring [18,19].

### Practical Implications of the Results

The study highlights several challenges related to the quality of antenatal consultations in the Malemba Nkulu health zone. These findings underscore the need for concrete interventions to improve the effectiveness of maternal care and reduce maternal and neonatal mortality. In particular, we highlight 4 measures that may contribute to a lasting improvement in the care of pregnant women and a significant reduction in maternal morbidity and mortality:

1. Capacity building for service providers: this involves organizing continuing education for prenatal care providers, with an emphasis on diagnostic examinations and management protocols, as well as establishing a skills assessment system, ensuring compliance with WHO recommendations.
2. Improving access to prenatal care: this involves developing community programs to raise awareness among pregnant women about the importance of early monitoring and reducing financial barriers by subsidizing essential medical consultations and examinations.
3. Strengthening medical infrastructure: this involves ensuring the availability of medical equipment in health facilities and implementing a policy for the distribution of essential medicines for the prevention and management of maternal complications.
4. Monitoring and evaluation of interventions: this involves integrating a monitoring system to measure the impact of improvements on prenatal consultations and encouraging further studies to refine strategies and adjust maternal health policies.

### Limitations and Potential Sources of Bias

This study has several methodological limitations that must be taken into account.

#### Selection Bias

The sample studied is based on pregnant women who consulted in the 8 selected health structures, which may introduce a representativeness bias. In particular, this study could overestimate or underestimate the quality of prenatal care depending on the specific characteristics of the institutions selected. Notably, however, the magnitude of selection bias is low since there was no stratification that could have favored certain subgroups. This means that the results are generally representative of the study population, although random variations may remain.

#### Information Bias

Some variables, including the pregnant women’s medical history and provider protocols, are based on medical records and self-reports from pregnant women; therefore, there is a risk of overestimating compliance with care, as some information may be incomplete or influenced by recall bias. We consider the magnitude of bias to be moderate because the data were collected by direct observation and validation of medical records, reducing potential errors.

#### Missing Data and Statistical Treatment

Although imputation methods were used, some missing data regarding laboratory tests and provider training could affect the accuracy of the results, leading to potential overestimation or underestimation of the quality of services based on missing data. We consider the magnitude of bias to be low to moderate as sensitivity analyses confirmed the robustness of the results.

Despite these limitations, this study provides a vital assessment of the quality of prenatal consultations and highlights priority areas for improvement. However, further research, particularly longitudinal, would be needed to refine the analysis and strengthen the generalizability of the results.

#### Generalizability of the Study Results

The study sample was selected by simple random sampling, ensuring an equal probability of inclusion for each patient. This methodological choice strengthens external validity, as it limits selection bias and improves the ability to generalize the results to all pregnant women attending health facilities in the Malemba Nkulu health zone. However, some constraints may affect generalizability. For example, the sample size, although statistically sufficient for analysis, might not capture all the nuances present in the entire target population. Additionally, women who did not attend the health facilities studied were excluded, which limits the applicability of the results to pregnant women who do not have access to prenatal care. The results obtained are consistent with similar studies conducted in sub-Saharan Africa, notably in Nigeria, Ghana, Rwanda, and Ethiopia, where comparable problems have been observed in terms of access to prenatal care and the training of providers.

Specific local factors may also limit the generalizability of the results, such as structural differences in the organization of care (some countries have implemented targeted reforms, significantly improving access to prenatal care) and variability of prenatal monitoring protocols (national recommendations can influence the application of quality standards).

The issues identified in this study, notably, the limits of training providers in environments where traditional midwives play a dominant role and financial and geographical barriers that restrict access to early prenatal consultations, are broadly representative of the challenges encountered in several health zones in the DRC. However, local adaptations would be necessary to replicate these findings in urban contexts, where medical infrastructure is more developed and pregnant women have better access to specialized care.

The external validity of this study is moderately high, as simple random sampling enhances the representativeness of the results. However, certain contextual specificities must be taken into account before extrapolating these findings to other regions of the DRC or to other African countries.

### Conclusions

This study reveals the poor quality of ANC1s in the Malemba Nkulu health zone, with only 2% meeting established standards. The findings underscore critical gaps in provider training, limited access to essential diagnostic tests, and insufficient awareness among pregnant women regarding the importance of early and comprehensive prenatal care. Addressing these deficiencies requires targeted interventions to strengthen health worker capacity, improve service availability, and enhance community engagement in maternal health.

Among the challenges identified, 43% of consultations were conducted by midwives without advanced medical qualifications, compromising the quality of follow-up. Furthermore, 52.4% of women had their ANC1 in the third trimester, limiting the impact of preventive measures.

To improve the quality of prenatal care, several recommendations can be made, including (1) strengthening the training of service providers through practical sessions and adapted certifications, (2) subsidy for prenatal services to ensure better access to care, (3) community awareness to encourage early consultations, and (4) integration of prenatal care into maternal health programs. These measures could contribute to a significant reduction in maternal morbidity and mortality. Further research is needed to evaluate the impact of the proposed interventions and ensure effective management.

## Abbreviations

ANC: antenatal care
ANC1: first antenatal consultation
DRC: Democratic Republic of Congo
WHO: World Health Organization
